# A Genetically-Engineered Thyroid Gland Built for Selective Triiodothyronine Secretion

**DOI:** 10.3390/ijms26157166

**Published:** 2025-07-24

**Authors:** Cintia E. Citterio, Berenice Morales-Rodriguez, Xiao-Hui Liao, Catherine Vu, Rachel Nguyen, Jessie Tsai, Jennifer Le, Ibrahim Metawea, Ming Liu, David P. Olson, Samuel Refetoff, Peter Arvan

**Affiliations:** 1 Department of Biomedical and Pharmaceutical Sciences, School of Pharmacy, Chapman University, Irvine, CA 92618, USA; 2 Department of Medicine, The University of Chicago, Chicago, IL 60637, USA; 3 Division of Metabolism, Endocrinology & Diabetes, Department of Internal Medicine, University of Michigan, Ann Arbor, MI 48109, USA; 4 Division of Endocrinology and Metabolism, Department of Internal Medicine, Tianjin Medical University General Hospital, Tianjin 300052, China; 5 Department of Pediatrics, University of Michigan, Ann Arbor, MI 48109, USA; 6 Department of Medicine and Pediatrics and Committees on Genetics, The University of Chicago, Chicago, IL 60637, USA

**Keywords:** thyroid, thyroid hormones, triiodothyronine, thyroglobulin

## Abstract

Thyroid hormones (thyroxine, T_4_, and triiodothyronine, T_3_) are indispensable for sustaining vertebrate life, and their deficiency gives rise to a wide range of symptoms characteristic of hypothyroidism, affecting 5–10% of the world’s population. The precursor for thyroid hormone synthesis is thyroglobulin (Tg), a large iodoglycoprotein consisting of upstream regions I-II-III (responsible for synthesis of most T_4_) and the C-terminal CholinEsterase-Like (ChEL) domain (responsible for synthesis of most T_3_, which can also be generated extrathyroidally by T_4_ deiodination). Using CRISPR/Cas9-mediated mutagenesis, we engineered a knock-in of secretory ChEL into the endogenous *TG* locus. Secretory ChEL acquires Golgi-type glycans and is properly delivered to the thyroid follicle lumen, where T_3_ is first formed. Homozygous knock-in mice are capable of thyroidal T_3_ synthesis but largely incompetent for T_4_ synthesis such that T_4_-to-T_3_ conversion contributes little. Instead, T_3_ production is regulated thyroidally by thyrotropin (TSH). Compared to *cog/cog* mice with conventional hypothyroidism (low serum T_4_ and T_3_), the body size of ChEL-knock-in mice is larger; although, these animals with profound T_4_ deficiency did exhibit a marked elevation of serum TSH and a large goiter, despite normal circulating T_3_ levels. ChEL knock-in mice exhibited a normal expression of hepatic markers of thyroid hormone action but impaired locomotor activities and increased anxiety-like behavior, highlighting tissue-specific differences in T_3_ versus T_4_ action, reflecting key considerations in patients receiving thyroid hormone replacement therapy.

## 1. Introduction

In vertebrates, the zero thyroid hormone is ultimately incompatible with life [1,2]. Throughout the evolution of vertebrates dating ~500 million years, iodinated glycoprotein thyroglobulin (Tg) is the only known precursor for thyroid hormone synthesis [3,4]. Tg is a multidomain secretory protein composed of upstream regions I-II-III responsible for the vast majority of thyroxine (T_4_) production and the C-terminal CholinEsterase-Like (ChEL) domain responsible for the majority of thyroidal triiodothyronine (T_3_) synthesis [3,5,6,7,8,9]. Tg is synthesized in the thyrocyte endoplasmic reticulum (ER), where it acquires N-linked glycosylation and conformational maturation [3,10]. En route to the thyroid follicular lumen, Tg acquires Golgi modifications of its N-glycans, which converts it from an endoglycosidase (endo) H-sensitive form to an endo H-resistant form [4]. Within the follicle lumen, selected Tg-tyrosine residues undergo iodination and coupling [7,8], leading to the formation of T_4_ (primarily within upstream regions I-II-III) [3,5,6,8,9] and the de novo synthesis of T_3_ (predominantly in the C-terminal ChEL domain) [3,7,8,11]. Endocytosis of Tg from the follicle lumen, followed by its lysosomal digestion, releases thyroid hormones for their transport to the bloodstream [3,12].

The thyroid gland normally provides 100% of the body’s daily supply of T_4_ but only a fraction of the daily supply of T_3_, with the remaining T_3_ coming from the extrathyroidal 5′-deiodination of T_4_ [by type-1 and -2 deiodinases (D1/D2) [3,13]]. Importantly, these deiodinases do not work on Tg as a substrate [11,14]. Although the fraction of circulating T_3_ provided by the normal thyroid gland varies in different species [~55% in rodents [15]], rodents and humans share a comparable regulation of the pituitary–thyroid axis [16,17]. Additionally, in both rodents and humans, thyroidal T_3_ increases when thyrotropin (TSH) receptors are stimulated by TSH [11,18]. Notably, mice lacking both D1 and D2 (D1/D2-KO) still produce normal levels of circulating T_3_ that come directly from the thyroid gland (in addition to abundant T_4_), driven by TSH stimulation [13,19].

Hypothyroidism, affecting 5–10% of the world’s population, is in most cases a lifelong condition [20], and is the most common congenital endocrinopathy [21]. Mutations altering the *TG* coding sequence are among the various genetic causes of the disease [22]. Such mutations trigger Tg misfolding [leading to a dysfunctional protein [3,23,24,25]], as has been well described in homozygous *cog/cog* mice expressing Tg p.L2263P [26,27]. The stimulatory TSH response to hypothyroidism can lead to thyroid overgrowth (goiter), whereas the deficiency of the thyroid hormone itself is associated with impaired developmental body growth, metabolic defects, and neurological impairments, including both autonomic dysfunction and motor activity in humans and rodents [28,29].

Interestingly, hypothyroid patients treated with T_4_ monotherapy (thus, not replacing thyroidal T_3_) often have lower serum T_3_ levels than patients with an endogenous thyroid function, and many such patients suffer persistent hypothyroid symptoms [30,31,32]. Treating patients with T_3_ alone as a hormone replacement therapy might offer therapeutic value, but due to its short half-life in the circulation, it may lead to wide daily fluctuations in serum T_3_ ranging from symptomatically high to low levels [33,34,35]. In principle, in the absence of Graves’ disease or other causes of hyperthyroidism, excess serum T_3_ is avoided when it is generated endogenously. Thus, we are interested in characterizing the physiologic consequences of the endogenous production of (normal levels of) serum T_3_ from a thyroid gland that is incompetent for the production of T_4_.

In vitro studies have demonstrated that the secretory ChEL domain of Tg (immediately following a signal peptide) readily undergoes secretion, iodination, and T_3_ generation [36,37]. Here, we have created a novel knock-in mouse (named ChEL-KI) whose thyroid glands express secretory ChEL, replacing *TG* in the endogenous chromosomal *TG* locus. We show that unlike previously described *TG* mutations that impair protein folding in the ER, ChEL-KI animals physiologically regulate the production of secretory ChEL, which folds well and does not generate ER stress, and is properly delivered to the thyroid follicle lumen for T_3_ hormonogenesis, while the animals remain impaired in T_4_ hormonogenesis. In these T_4_-deficient animals, we characterize the selective impact of physiological levels of T_3_ unencumbered by wide pharmacological fluctuations that occur upon pharmacologic T_3_ replacement.

## 2. Results

### 2.1. Generation of ChEL-KI Mice and Characterization of the Trafficking of Secretory ChEL In Vivo

**Figure 1:**
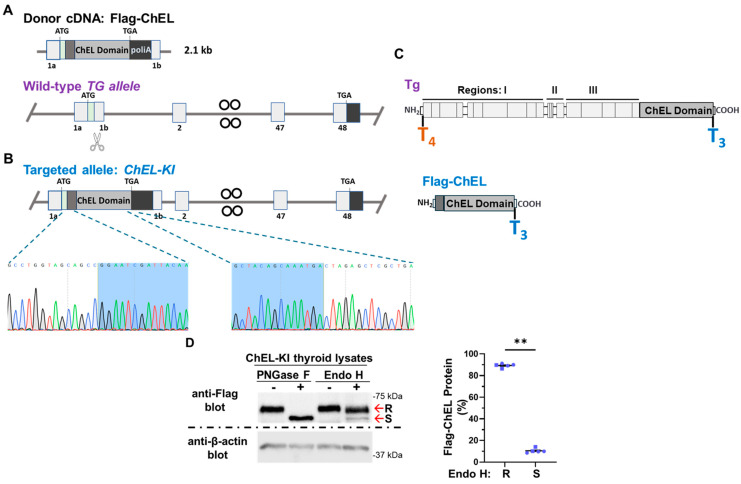
Generation of ChEL-KI mice and characterization of trafficking of secretory ChEL in vivo. (**A**) Schematic illustration of CRISPR/Cas9-mediated gene editing to knock-in *TG* within the exon 1 of the mouse *TG* gene. Both the donor cDNA encoding the N-terminal Flag-tagged ChEL (~2.1 kb) and the target site of CRISPR-Cas9 (scissors) in the wild-type *TG* allele are represented. *TG* exon numbers are shown below each exon (light-gray box). The *TG* signal peptide is represented by a light-green box. The poliA signal is denoted by a black box. The start codon (ATG) and stop codon (TGA) are indicated. (**B**) DNA sequences of the 5′ and 3′ arms of the ChEL-KI allele. Partial coding sequences of the Flag-ChEL are indicated in blue. (**C**) Schematic illustration of mature Tg and Flag-ChEL primary structures. The main hormonogenic sites within Tg, and Y25 and Y2764 are indicated in orange and blue, respectively. (**D**) SDS-Polyacrylamide gel electrophoresis of thyroid homogenates from female ChEL-KI mice previously treated (+) or untreated (−) with PNGase F or endoglycosidase H (endo H), followed by immunoblotting with the monoclonal anti-Flag antibody and monoclonal anti-β-actin antibody as indicated. Red arrows point at endo H-resistant (R) or endo H-sensitive (S) content within Flag-ChEL. Graph shows quantitation of endo H R content as well as the endo H S content in Flag-ChEL from five independent thyroid homogenates from ChEL-KI mice; mean ± SD; ** *p* < 0.01 (unpaired two-tailed Student’s *t*-test). Each dot represents an individual animal, with circles representing females and squares representing males.

ChEL-KI mice express secretory ChEL (epitope-tagged with Flag) in place of Tg. To achieve this, mouse Flag-ChEL cDNA was positioned immediately downstream of the endogenous *TG* signal peptide and followed by a stop codon and a strong polyadenylation signal (Figure 1A,B), which eliminates most sites of T_4_ formation (Figure 1B and Figure 1C lower panel, respectively). A ChEL-KI founder backcrossed to the C57BL6J strain confirmed germline delivery, with progeny then bred to homozygosity. During the propagation of homozygous mice, we provided T_4_ supplementation to pregnant females with suckling pups, with no further supplementation after weaning. Homozygous ChEL-KI mice lived to adulthood without thyroid hormone supplementation.

Thyroid tissues of adult ChEL-KI animals were digested with endo H to assess the intracellular trafficking of Flag-ChEL (64 kDa) from the ER to Golgi compartment, based on the status of processing of its N-linked glycans [38] (Supplemental Figure S1). Whereas PNGase F removed all N-linked oligosaccharides (a positive control), secretory ChEL was ~90% endo H-resistant (Figure 1D), indicating its efficient intracellular folding and export from the ER in vivo. The presence of the endo H-sensitive band (bearing only high-mannose-type N-glycans) is consistent with the ongoing thyroidal synthesis of the secretory ChEL protein in the ER. This fractional distribution of endo H-resistant and sensitive forms closely resembles that of the full-length wild-type (WT) Tg in the mouse thyroid gland [39] (Supplemental Figure S2).

### 2.2. Body Weight and Length of ChEL-KI Mice

**Figure 2:**
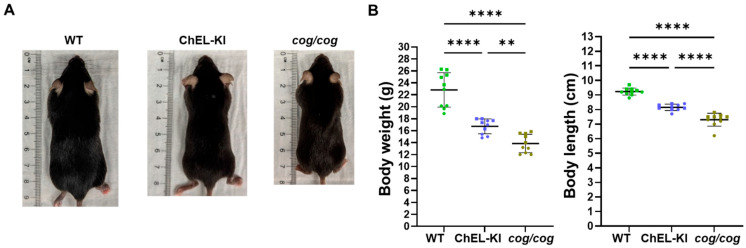
Body weight and length of ChEL-KI mice. (**A**) Representative images depicting body size of ~3-month-old male ChEL-KI mice, hypothyroid *cog/cog* mice, and euthyroid (wild-type, WT) controls. (**B**) Body weight and length of ~3-month-old ChEL-KI mice, hypothyroid *cog/cog* mice, and WT controls (*n* = 10 mice per group). Graphs show mean ± SD; ** *p* < 0.01, **** *p* < 0.0001 (one-way ANOVA with Tukey’s post hoc test). Each dot represents an individual animal, with circles representing females and squares representing males.

In early post-natal life, thyroid hormone production affects body growth. Compared to age-matched “conventionally hypothyroid” *cog/cog* mice [26,27], the body weight and length of adult homozygous ChEL-KI mice were significantly greater (Figure 2, one-way ANOVA, *p* < 0.01 and *p* < 0.0001, respectively), seen clearly in 6-week-old mice (Supplemental Figure S3, two-way ANOVA, *p* < 0.05 and *p* < 0.0001, respectively). Nevertheless, both groups of animals were smaller than the WT control mice that maintained normal serum T_4_ and T_3_ levels (Figure 2, one-way ANOVA, *p* < 0.0001 for each comparison). The body growth phenotype generally correlated with average serum growth hormone (GH) levels measured in 3-month-old mice, although the data did not achieve statistical significance (Supplemental Figure S4, one-way ANOVA, *p* > 0.05).

### 2.3. Thyroid Hormone Levels and Thyroid Gland Size in ChEL-KI Animals

**Figure 3:**
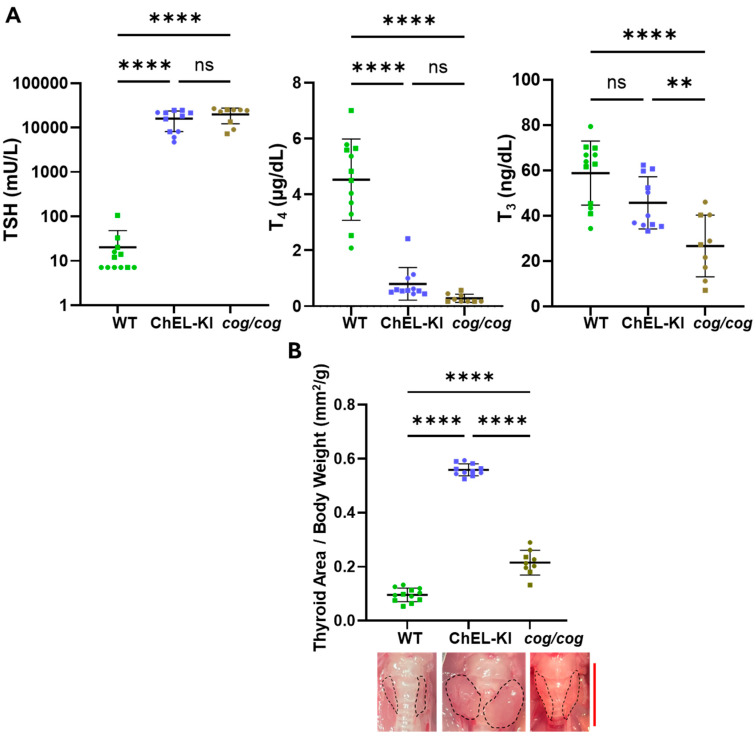
Thyroid status of ChEL-KI mice. (**A**) Serum levels of TSH and total T_4_ and T_3_ in 3-month-old ChEL-KI mice, hypothyroid *cog/cog* controls, and euthyroid (wild-type, WT) controls, as determined by RIA (*n* = 9–12 mice per group). In the graph showing serum TSH levels (first panel), the lower half of the error bar for WT was not plotted on the logarithmic *Y*-axis due to values approaching the negative range. (**B**) Thyroid gland size normalized to body weight in 3-month-old ChEL-KI mice, hypothyroid *cog*/*cog* mice, and WT controls (*n* = 9–12 mice per group). Representative thyroid glands from female mice are shown in the lower panel; the thyroid lobes are marked by dotted lines. Scale bar, 0.5 cm. Graphs show mean ± SD; ns, not significant (*p* > 0.05); ** *p* < 0.01, **** *p* < 0.0001 (1-way ANOVA with Tukey’s post hoc test). Each dot represents an individual animal, with circles representing females and squares representing males.

Adult homozygous ChEL-KI mice exhibited significantly increased serum TSH levels and extremely low total T_4_ (Figure 3A, first two panels, one-way ANOVA, *p* < 0.0001 and *p* < 0.0001, respectively). However, the average serum T_3_ level in ChEL-KI mice was significantly greater than that of “conventionally hypothyroid” *cog/cog* mice (that have dramatic lowering of both serum T_4_ and T_3_), and was actually not significantly lower than that of WT animals (Figure 3A, third panel, one-way ANOVA, *p* < 0.01 and *p* > 0.05, respectively). Interestingly, despite a similar increase in serum TSH to that of “conventionally hypothyroid” *cog/cog* mice (Figure 3A, first panel, one-way ANOVA, *p* > 0.05), the ChEL-KI mice with higher serum T_3_ levels grew a much larger goiter (Figure 3B, one-way ANOVA, *p* < 0.0001), suggesting an improved thyroid proliferative response to TSH stimulation.

### 2.4. Characterization of the Thyroid Gland in ChEL-KI Mice

**Figure 4:**
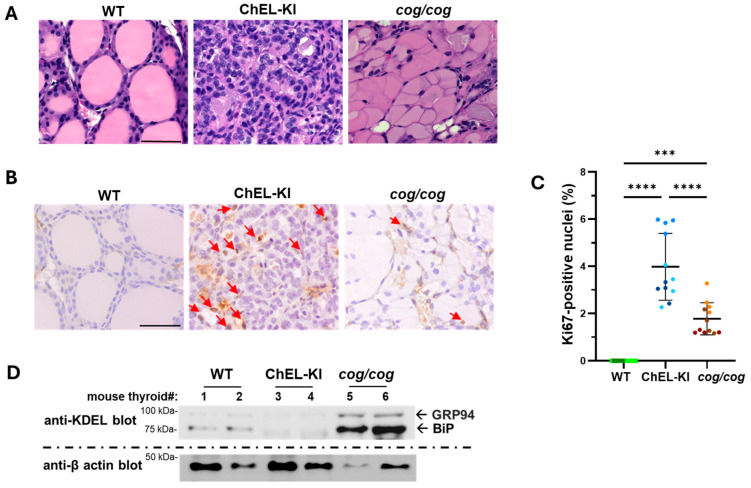
Histological characteristics of the thyroid gland in ChEL-KI mice. (**A**) Representative H&E staining of thyroid glands from 3-month-old female ChEL-KI mice, hypothyroid *cog*/*cog* mice, and wild-type (WT) controls, showing smaller colloids in ChEL-KI mice and classical thyrocyte distention in *cog*/*cog* mice. The scale bar represents 50 μm. (**B**) Ki67 immunohistochemistry of the thyroid gland from age-matched female ChEL-KI, *cog*/*cog*, and WT mice. Red arrows indicate Ki67-positive nuclei. The scale bar represents 50 μm. (**C**) Quantification of images like those shown in panel B presented as Ki67-positive nuclei as a proportion of total nuclei in thyroid images (*n* = 3 animals per group; each color represents a single animal; each point is an independent section; circles represent females and squares represent males); *** *p* < 0.001, **** *p* < 0.0001 (one-way ANOVA with Tukey’s post hoc test). (**D**) SDS-Polyacrylamide gel electrophoresis of thyroid homogenates from 3-month-old ChEL-KI mice, *cog*/*cog* mice, and WT animals, followed by immunoblotting with a mAb anti-KDEL and a mAb anti-β-actin as indicated. KDEL-containing ER chaperones GRP94 and BiP are indicated with black arrows. Thyroids 1, 3, and 5 were obtained from females; 2, 4, and 6 from males. The immunoblot was repeated three times using independent samples, consistently reproducing this result.

Adult ChEL-KI mouse thyroid glands exhibited small thyroid follicular lumina (Figure 4A), consistent with active ongoing TSH stimulation [40]. Accompanying the increase in hematoxylin-stained nuclei (Figure 4A) and larger goiters in ChEL-KI animals (Figure 3B), thyroid cell proliferation by Ki67 immunostaining was increased in ChEL-KI mice compared to WT and *cog*/*cog* animals (Figure 4B,C, one-way ANOVA, *p* < 0.0001 and *p* < 0.0001, respectively). While the histology of *cog*/*cog* mouse thyroids showed a large distension of the thyrocyte cytoplasm (comprised largely of ER containing misfolded mutant Tg) as previously described [39], such cytoplasmic distension was not observed in the thyroid glands of ChEL-KI mice (Figure 4A). Additionally, KDEL-containing proteins (primarily ER chaperones) were not increased in ChEL-KI thyroid tissue (Figure 4D); thus, unlike other forms of mutant Tg protein [3,23,24,25], secretory ChEL does not elicit an ER stress response.

**Figure 5:**
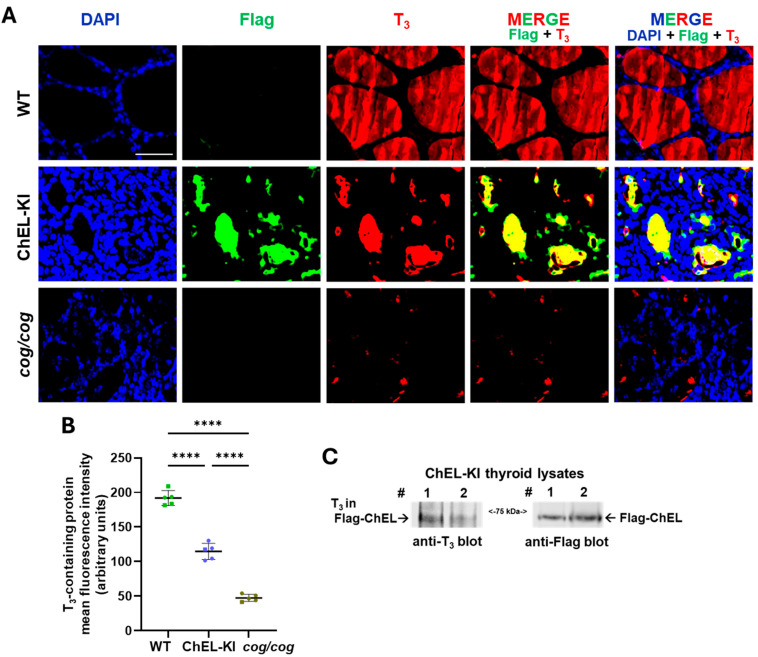
T_3_-containing protein in the thyroid gland of ChEL-KI mice. (**A**) Immunofluorescence of T_3_-containing protein (red) and Flag-ChEL (green) in the thyroid of female ChEL-KI mice, with DAPI counterstaining (blue). Age-matched female *cog*/*cog* and wild-type (WT) mice were used as controls. Localization of T_3_-containing protein (red) and Flag-ChEL (green) to the follicular lumen is evident in the thyroid gland of ChEL-KI mice, MERGE panels. The scale bar represents 50 μm. (**B**) Graph shows the quantification of the mean fluorescence intensity of the T_3_-containing protein. Data are expressed as mean ±  SD; *n* = 5 animals per group; ns, not significant; **** *p* < 0.0001 (one-way ANOVA with Tukey’s post hoc test). Each dot represents an individual animal, with circles representing females and squares representing males. (**C**) SDS-Polyacrylamide gel electrophoresis of thyroid homogenates from ~3-month-old ChEL-KI mice followed by immunoblotting with a mouse anti-T_3_ monoclonal antibody and a rabbit anti-Flag monoclonal antibody as indicated. T_3_ in Flag-ChEL is indicated on the left panel. Thyroid # 1 was obtained from a female, and # 2 from a male. These results were reproduced in three independent experiments.

Immunofluorescent staining of Flag-ChEL and T_3_-containing protein indicated that most Flag-ChEL in ChEL-KI mouse thyroids is not trapped in the ER but resides in the follicle lumina where the T_3_-containing protein is localized (Figure 5A). Immunoblotting of thyroid lysates from ChEL-KI mice detected a T_3_-containing protein at the precise position of Flag-ChEL (Figure 5C). Importantly, the thyroid glands of ChEL-KI mice exhibited a higher signal for intrathyroidal T_3_-containing protein than that observed in *cog/cog* mice (Figure 5A,B). Moreover, despite a ~40% decrease in the signal per unit area for thyroidal T_3_-containing protein compared to that of WT animals (Figure 5B, one-way ANOVA, *p* < 0.0001), when accounting for the ~6-fold enlargement of the thyroid area in ChEL-KI mice (Figure 3B), the data suggest that total number of thyroidal T_3_-containing protein in TSH-stimulated ChEL-KI mice (that are severely deficient of T_4_ and thus must make most of their T_3_ within the thyroid gland itself) is roughly 3.6-fold greater than that in WT animals (which make much of their T_3_ extrathyroidally, by T_4_ deiodination).

### 2.5. Evidence of T_3_ Action in ChEL-KI Mice

**Figure 6:**
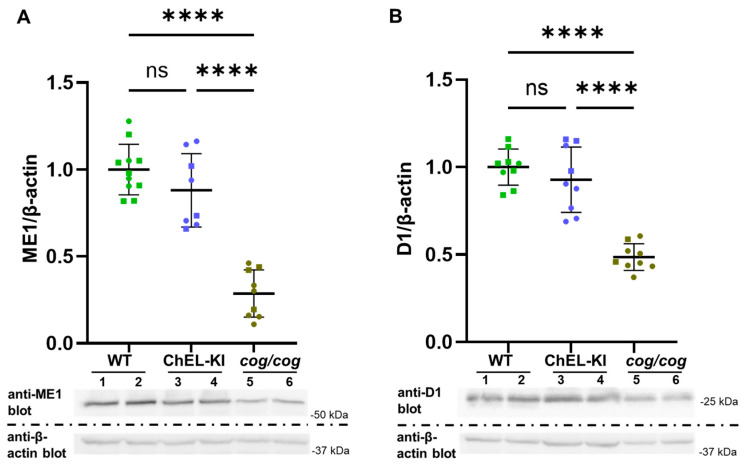
Expression of markers of T_3_ action in the liver of ChEL-KI mice. (**A**) Expression of malic enzyme 1 (ME1). Lower panel: SDS-polyacrylamide gel electrophoresis of liver homogenates from 3-month-old ChEL-KI mice, *cog*/*cog* animals, and wild-type (WT) mice, followed by immunoblotting with anti-ME1 antibodies and anti-β-actin antibodies as indicated. Upper panel: Graph shows the densitometric quantitation of hepatic ME1 protein expression normalized to β-actin. (**B**) Expression of type-1 deiodinase (D1). Lower panel: SDS-polyacrylamide gel electrophoresis of liver homogenates from ChEL-KI mice, *cog*/*cog* animals, and WT controls, followed by immunoblotting with anti-D1 antibodies and anti-β-actin antibodies as indicated. Upper panel: Graph shows the densitometric quantitation of hepatic D1 protein expression normalized to β-actin. Each dot represents an individual animal, with circles representing females and squares representing males; *n* = 8–11 animals per group; mean ±  SD; ns, not significant (*p* > 0.05), *p*; **** *p* < 0.0001 (one-way ANOVA with Tukey’s post hoc test).

Homozygous 3-month-old ChEL-KI and *cog/cog* mice both exhibit extremely low serum T_4_ levels but differ significantly in serum T_3_ levels (Figure 3A). With this in mind, we analyzed the protein expression of the hepatic malic enzyme (ME1 64 kDa) and D1 (29 kDa), both of which have been reported to be upregulated by T_3_ [41,42]. Western blots of liver homogenates revealed normal expression levels of ME1 and D1 in ChEL-KI animals, whereas *cog*/*cog* mice (with abnormally low serum T_3_) expressed a significant decrease in the level of both proteins (Figure 6A,B, respectively; one-way ANOVA, *p* values are indicated in the legend in Figure 6).

### 2.6. Behavior of ChEL-KI Animals

**Figure 7:**
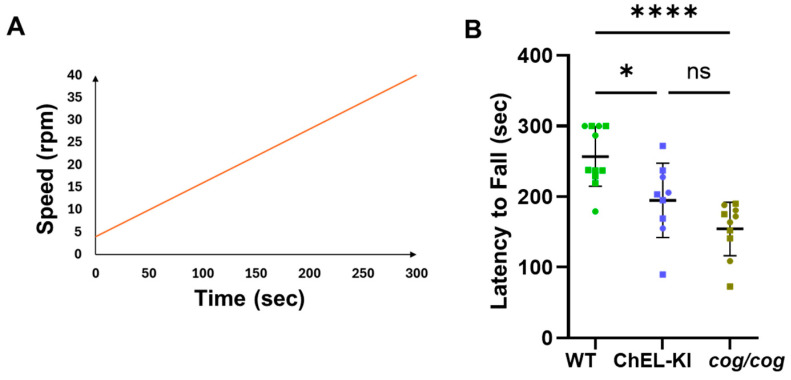
Motor activity assessment of ChEL-KI mice. (**A**) Schematic representation illustrating the settings used for the accelerating speed test: the rotarod was set to accelerate from 4 to 40 rpm (shown on the *y*-axis) over a maximum of 300 s (shown on the *x*-axis). (**B**) Latency to fall from the accelerating rotarod in adult ChEL-KI mice, age-matched *cog*/*cog* mice, and wild-type (WT) animals (*n* = 9–11 animals per group). Each dot represents an individual animal, with circles representing females and squares representing males. One-way ANOVA with Tukey’s post hoc test; mean ±  SD; ns, not significant (*p* > 0.05); * *p* < 0.05, **** *p* < 0.0001.

The CNS is known to use a D2-mediated deiodination of T_4_ to generate T_3_ in various brain regions [13,43]. Because ChEL-KI and *cog/cog* mice both offer extremely low serum T_4_ levels to the brain, it was of interest to examine the behavioral performance in these two animal models of hypothyroidism that are distinguished by differences in serum T_3_. The rotarod accelerating test (Figure 7A), which examines motor activity and coordination, revealed that adult WT animals were able to stay on the device longer than either ChEL-KI or *cog*/*cog* mice (Figure 7B, one-way ANOVA, *p* < 0.05 and *p* < 0.0001, respectively). Average latency-to-fall appeared worse in *cog*/*cog* mice, but this was not statistically different from that of ChEL-KI mice, suggesting that differences between the two hypothyroid genotypes were at most modest (Figure 7B, one-way ANOVA, *p* > 0.05).

**Figure 8:**
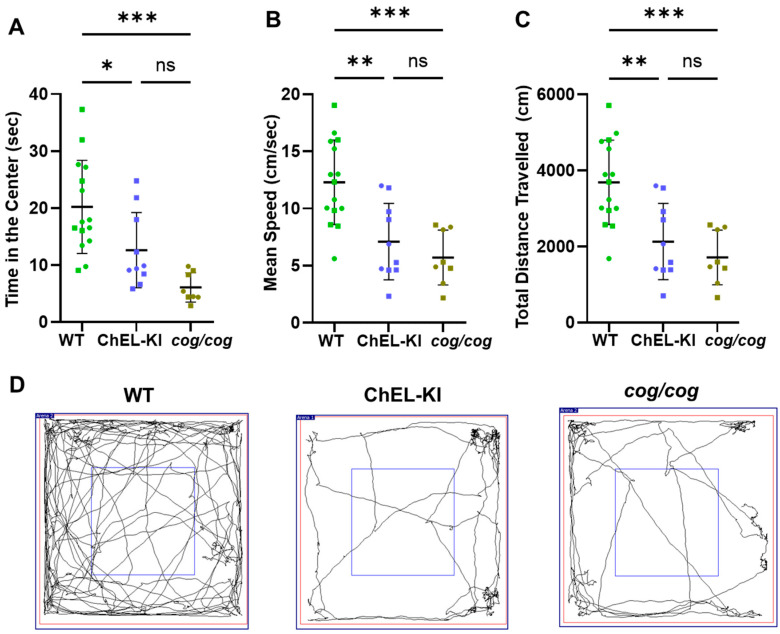
Anxiety-like behavior and locomotor activity levels in ChEL-KI mice. (**A**) Time spent in the center zone of the arena, (**B**) overall mean speed, and (**C**) total distance traveled during the 300 s open-field test by adult ChEL-KI mice, age-matched *cog*/*cog* animals, and wild-type (WT) mice. *n* = 8–15 animals per group. Each dot represents an individual animal, with circles representing females and squares representing males. One-way ANOVA with Tukey’s post hoc test; mean ±  SD; ns, not significant (*p* > 0.05); * *p* < 0.05, ** *p* < 0.01, *** *p* < 0.001. (**D**) Representative trajectory maps showing the movement of female ChEL-KI mice, *cog*/*cog* mice, and WT animals. The arena is delimited in red. The small blue square delimits the center of the arena.

The open-field test assesses both locomotor and anxiety-related activity. Time spent in the center of the arena (an inverse measure of anxiety-like behavior) was significantly reduced in both ChEL-KI and *cog*/*cog* mice (Figure 8A, one-way ANOVA, *p* < 0.05 and *p* < 0.001, respectively; and Figure 8D). Hypothyroid *cog/cog* mice tended to spend even less time in the center, but again the difference was not statistically different from that of ChEL-KI (Figure 8A, one-way ANOVA, *p* > 0.05). Finally, the mean speed and total distance traveled were decreased in both ChEL-KI and *cog*/*cog* mice (Figure 8B,C, one-way ANOVA, *p* < 0.01 and *p* < 0.001, respectively); once again, hypothyroid *cog*/*cog* mice tended to be even worse, but with results that did not achieve statistical significance (Figure 8B,C, one-way ANOVA, *p* < 0.05). Thus, for these behavioral phenotypes, the benefit of a selectively improved serum T_3_ level in ChEL-KI mice appeared limited.

## 3. Discussion

In the present study, we genetically-engineered mice with a thyroid gland built for the selective generation of T_3_ in order to better segregate T_4_- and T_3_-dependent phenotypes at the whole-body level. Homozygous ChEL-KI mice with a selective deficiency of serum T_4_ were compared to “conventionally hypothyroid” cog/cog animals that suffered from both low circulating T_4_ and T_3_. It could be argued that it would be better to use conventionally hypothyroid mice that have little or no serum T_4_ and T_3_, or animals (or humans) completely lacking a thyroid gland, in order to study the effects of selective supplementation with exogenous T_4_ or T_3_. Indeed, such studies have been reported [44,45,46] and they have considerable value—but it has been virtually impossible to maintain long-term serum T_3_ steadily in the physiologic range using simple pharmacologic replacement doses [32,47], which may be a confounding variable.

Although the ChEL-KI mouse thyroid is incompetent for T_4_ formation, Flag-ChEL allows for preservation of the main T_3_-formation site on Tg (Figure 1A–C). Unlike other mouse models bearing *TG* mutations [3,23,24,25], ChEL-KI mice thyroidally do not express a Tg variant that misfolds (Figure 1D) or brings about cytoplasmic swelling in the thyrocyte, or activates an ER stress response (Figure 4A,D). Indeed, anterograde Flag-ChEL trafficking through the thyrocyte secretory pathway appears equally efficient to that of full-length Tg, as judged by the acquisition of endo H-resistance (Figure 1D and Supplemental Figure S2). After export from the ER to the Golgi complex, Flag-ChEL reaches the thyroid follicle lumen where T_3_ is formed (Figure 5). Selective T_4_ deficiency limits the substrate for T_4_-to-T_3_ conversion, but in ChEL-KI mice this is largely compensated by an increase in the formation of T_3_-containing protein within the thyroid gland (Figure 5A,B).

The increase in thyroidal T_3_-containing protein leading to near-normal circulating T_3_ levels requires ongoing stimulation by TSH, which acts in several important ways. First, TSH stimulates the endocytosis of iodoproteins from the thyroid follicle lumen leading to lysosomal proteolysis, thus enhancing thyroid hormone release to the bloodstream [12,48,49]. Enhanced endocytic activity is consistent with the smaller thyroid follicles observed in TSH-stimulated ChEL-KI mice (Figure 4A). Second, TSH promotes the growth of the entire thyroid gland [50,51] (discussed below). And third, TSH stimulation enhances de novo T_3_ synthesis in the ChEL domain [11,36]. These effects, together, can explain the near-normal levels of T_3_ measured in the circulation of homozygous ChEL-KI mice (Figure 3A, third panel).

Hypothyroid mice and humans can exhibit stunted growth, partially from the loss of direct thyroid hormone effects on the skeleton, but also caused by a reduction in GH levels [52,53]. ChEL-KI mice with largely preserved serum T_3_ exhibited a larger body length and heavier weight than “conventionally hypothyroid” *cog*/*cog* mice, but still remained substantially below normal (Figure 2). We cannot completely exclude the possibility that a tiny difference in serum T_4_ between ChEL-KI and *cog*/*cog* mice (Figure 3A, middle panel) might make a contribution to differences in body growth, but serum T_4_ is not statistically different between the two genotypes. Plausibly, diminished serum T_4_ may result in reduced GH expression/secretion from the anterior pituitary (although we were only able to examine serum GH levels at a single time point in a limited number of animals; Supplemental Figure S4). Further supporting a key role of T_4_ at the level of the anterior pituitary is the observed absence of the negative regulation of TSH expression/secretion in ChEL-KI animals despite normal serum T_3_ levels (Figure 3A)—highlighting the importance of local D2-mediated T_4_-to-T_3_ conversion [54,55]. Additionally, the preservation of serum T_3_ (Figure 3A, third panel) may contribute in part to the greater post-natal body growth in ChEL-KI animals than *cog*/*cog* animals (Figure 2).

The liver does not express D2 [56] and relies significantly on circulating T_3_ [46,57]. Our data (Figure 6) support the view that normal hepatic protein levels of ME1 and D1 do not require normal circulating levels of T_4_ but are sensitive to circulating levels of T_3._

In contrast with the liver, the CNS is thought to rely substantially on local T_3_ production from T_4_ via D2-mediated 5′-deiodination [13,43]. We examined additional CNS functions that have been reported to be linked to phenotypes in hypothyroid rodents and humans [29,58,59,60]. Both ChEL-KI and *cog*/*cog* mice exhibited behavioral abnormalities, such as impaired motor activity and locomotion, as well as increased anxiety-like behavior compared to euthyroid controls (Figure 7B and Figure 8). As the behavioral data show no statistical significance between ChEL-KI and *cog*/*cog* mice, we conclude that normal or near-normal circulating T_3_ cannot efficiently replace the role of T_4_ (and the local generation of T_3_ in the CNS) in supporting these normal behaviors. Our conclusion is consistent with the phenotype (s) observed in adult mice with D2 deletion [61,62].

Crucial to the generation of normal or near-normal serum T_3_ levels in ChEL-KI mice, despite the lack of T_4_ substrate for 5′-deiodination, was the efficient TSH-stimulated growth of a goiter (Figure 3). The reason why ChEL-KI mice grow an even larger goiter (Figure 3B) with increased cell proliferation (Figure 4B,C) compared to age-matched *cog*/*cog* mice with similarly high TSH levels (Figure 3A) remains to be elucidated. While TSH is crucial for regulating the size and function of the thyroid gland, extra-pituitary mechanisms may also contribute [63,64,65]. For example, T_3_ enhances TSH proliferative effects in cultured thyrocytes [66,67], although it has yet to be established whether this occurs in vivo. Nevertheless, the ability to form intrathyroidal T_3_-containing protein (Figure 5) highlights that the enlarged thyroid gland (Figure 3B) is a key compensatory response that helps to sustain normal or nearly-normal circulating T_3_ (Figure 3A, third panel) in homozygous ChEL-KI animals.

In summary, we genetically-engineered mice with a thyroid gland built for the selective generation of T_3_ from the T_3_-forming ChEL domain of Tg. These novel ChEL-KI mice exhibit significant T_4_ deficiency with high TSH and a large goiter, resulting in normal levels of circulating T_3_ as a biological necessity. This is the first animal model to reveal an in vivo role of one of Tg’s evolutionarily conserved domains [4,14] and also the first to examine the physiology of a thyroidally derived T_3_-centric hormonal environment. The endogenous circulating T_3_ in the near-absence of T_4_ can maintain life and rescue some hypothyroid phenotypes but not others, consistent with a complex interplay between circulating and locally produced T_3_ in sustaining thyroid hormone action in body tissues. The setting of low T_4_ and normal or nearly-normal serum T_3_ maintained by TSH stimulation and goiter has been reported in some human patients [68]. Careful human phenotyping of such patients may provide valuable insights into the role of specific thyroid hormone replacement therapies in correcting selective phenotypes. Further research of this kind can add to our understanding of the best personalized treatment strategies for different patients suffering from hypothyroidism.

## 4. Materials and Methods

### 4.1. Primary Antibodies

DYKDDDDK Tag rabbit mAb (D6W5B, Cell Signaling Technology, Danvers, MA, USA), rabbit monoclonal [EPR9730] to thyroglobulin Ab (Abcam, Cambridge, UK), T_3_ mouse mAb (3A6, Invitrogen, Waltham, MA, USA), DIO1 (B-7) mAb (sc-515198, Santa Cruz Biotechnologies, Dallas, TX, USA), ME1 (C-6) mAb (sc-365891, Santa Cruz Biotechnologies, Dallas, TX, USA), Ki67 mAb (ab16667, Abcam, Cambridge, UK), KDEL (10C) mAb (ADI-SPA-827-D Enzo Life Sciences, Long Island, NY, USA), anti-Flag M2 mAb (F1804, MilliporeSigma, Burlington, MA, USA), β-Actin (C4) mAb (sc-47778, Santa Cruz Biotechnologies, Dallas, TX, USA), and HSP 90α/β (F-8) mAb (sc-13119, Santa Cruz Biotechnologies, Dallas, TX, USA) were used.

### 4.2. Animals

To engineer the ChEL-KI mouse, we used CRISPR/Cas9-mediated mutagenesis creating a large edition in the endogenous mouse *TG* gene (ENSMUST00000065916.14, NM_009375.2→NP_033401.2) including all 5′ upstream regulatory elements yet encoding only the Tg-ChEL domain (Flag-tagged, immediately downstream of the endogenous Tg signal peptide, and followed by a stop codon and a strong polyadenylation signal), as shown in Figure 1A,B. Briefly, a CRISPR-Cas9 system, gRNA (5′-CTGGTAGCAGCCAACATCTT-3′), and donor Flag-ChEL-poliA cDNA (Figure 1A) were co-injected into the fertilized eggs of C57BL/6JGpt mice for homologous recombination, and the zygotes were transferred into the oviduct of pseudopregnant ICR females at 0.5 dpc. F0 offsprings were identified by PCR and DNA sequencing analyses. The stable, inheritable, positive F1 mouse model was created by mating F0 mice with wild-type mice. These were further bred with WT C57BL6J obtained from JAX. Then, heterozygous ChEL-KI mice were bred to homozygosity (and heterozygosity). T_4_ supplementation in drinking water (1 μg/mL, T2501, MilliporeSigma, Burlington, MA, USA) was provided to pregnant ChEL-KI homozygotes and females with suckling pups, only until the litter was 4 weeks old. WT animals were littermates in the same strain background. *cog*/*cog* (homozygous Tg p.L2263P) mice in the C57BL6J background [26,27] were used as hypothyroid controls. T_4_ supplementation in drinking water (1 μg/mL, T2501, MilliporeSigma, Burlington, MA, USA) was provided to pregnant *cog*/*cog* homozygotes and females with suckling pups, only until the litter was 4 weeks old. Mouse genotyping was performed either by end-point PCR with the set of primers indicated in Supplemental Table S1 followed by agarose gel separation of the PCR fragments with an adjacent DNA ladder, as in Supplemental Figure S5, or through real-time PCR by automated genotyping services. Both male and female animals were used. Mice were housed in an institutional facility, in individually ventilated cages, at 22 °C, with a 12 h dark/light cycle. Unless otherwise indicated, mice were 2.6–3.5-months old. The sex of each animal used in each of the experiments is identified in the graphs in Figure 1D, Figure 2B, Figure 3A,B, Figure 4C, Figure 5B, Figure 6A,B, Figure 7B, Figure 8A–C and Figures S2–S4; males are indicated by squares, females by circles.

### 4.3. Body Weight and Length Measurements

Mouse body weight and length were recorded weekly. The body length was measured from the nose tip to tail base, as shown in Figure 2.

### 4.4. Serum Hormone Measurements

Serum total T_4_, total T_3_, and TSH concentrations were measured using radioimmunoassays as previously described [69,70]. Serum growth hormone concentration was measured using the Mouse/Rat Growth Hormone ELISA kit, Catalog 22-GHOMS-E01, from ALPCO, Salem, NH, USA [71].

### 4.5. Thyroid Gland Size Measurement

Thyroids were dissected post-euthanasia with both lobes fully exposed. The areas of the thyroid glands were measured from dissection images containing an in situ calibrated size marker. The thyroid areas were quantified using ImageJ software, version 1.54m (NIH) and normalized to each animal’s body weight, as previously described [72].

### 4.6. Western Blotting

Mouse thyroid glands or left lobes of livers were lysed by sonication in RIPA buffer containing a protease inhibitor mixture. Lysates were cleared at 12,000× *g* for 10 min at 4 °C and total protein was determined by Bramhall assay [11,73]. Samples (5 μg of total protein per lane) were subjected to SDS-PAGE under reducing conditions. Pre-stained molecular mass markers were run in lanes adjacent to the experimental samples. Proteins were electrotransferred to nitrocellulose membranes, blocked with 5% milk in TBS plus 0.05% Tween 20 (TBS-T), immunoblotted with the indicated antibodies and appropriate horseradish peroxidase–conjugated secondary antibody, and visualized by enhanced chemiluminescence. For the T_3_ immunoblots, primary mouse mAb anti-T_3_ was diluted at 1:1000 containing 500 ng/mL of free T_4_ (to eliminate any possibility of T_4_ cross-reactivity) [11,36]. Images were captured in a ChemiDoc XRS+ system (Bio-Rad, Hercules, CA, USA). Endoglycosidase H and PNGaseF digestions were performed as previously described [72,74]. Band intensities were quantified using ImageJ [75].

### 4.7. Histology and Immunostaining of Thyroid Sections

Thyroids from mice were dissected, formalin-fixed, paraffin-embedded, sectioned (5 μm), deparaffinized in Xylene, and stained with hematoxylin–eosin (VectorLabs, Newark, CA, USA). Images were taken with a Keyence BZ-X710 microscope (Osaka, Japan). For immunofluorescence, thyroid sections were deparaffinized in Xylene, followed by antigen retrieval in citrate buffer (12.3 mM, pH 6), permeabilization with soaking buffer (0.4% Triton X-100 in TBS), and treated with blocking buffer (3% BSA TBS/0.2% TX-100 in TBS) at room temperature before incubation with primary antibodies overnight at 4 °C. A total of 500 ng/mL of free T_4_ was added to the mouse anti-T_3_ antibody solution as previously described [11]. After washing, the sections were then incubated with Alexa Fluor-conjugated secondary antibodies (Thermo Fisher Scientific, Waltham, MA, USA) for 1 h at room temperature. Sections were counterstained with Prolong-Gold and DAPI (Invitrogen) and imaged with a Nikon Ti-E confocal microscope (Tokyo, Japan). Immunofluorescence intensities were quantified using ImageJ utilizing the ROI manager function [76]. Four non-overlapping regions of interests (ROIs) of the same size were analyzed per mouse and averaged to obtain the mean fluorescence intensity. For immunohistochemistry, anti-Ki67 staining was performed as previously described [39]. Images were obtained using an Olympus CX23 microscope (40× objective, Tokyo, Japan). Analysis of four non-overlapping regions of the same size was performed per mouse slide, and Ki67-positive nuclei were quantified as a proportion of total nuclei.

### 4.8. Rotarod Motor Test

Prior to conducting the accelerating rotarod assay, the mice were pre-trained on the rotarod (Ugo Basile, Gemonio, Italy) twice per day for 3 consecutive days at a constant speed of 4 rpm, for up to a maximum of 120 s. For the accelerating speed test, the rotarod was set to accelerate from 4 to 40 rpm over 300 s, and the time until the mice fell from the rod was recorded (Figure 7A). The average of three accelerated speed test trials conducted on the same day was calculated and represented in Figure 7B. Both sexes were analyzed; in the graphs in Figure 7B, males are indicated by squares and females by circles.

### 4.9. Open-Field Behavioral Test

Locomotor activity and anxiety were assessed with the open-field behavioral test as described previously [77]. The mice were acclimatized for at least 30 min before the test and were then placed in an open box (72 cm × 72 cm with 36 cm walls) with a center square (36 cm × 36 cm). SMART Video Tracking Software, version 3.0 (Panlab, Harvard Apparatus, Holliston, MA, USA) tracked the mice for five min. Locomotor activity was evaluated by measuring the mean speed and total distance traveled by the mice. Anxiety was evaluated by measuring the amount of time the mice spent in the center. Both sexes were analyzed; in the graphs in Figure 8A–C, males are indicated by squares and females by circles.

### 4.10. Statistical Analysis

One-way ANOVA followed by Tukey’s multiple comparison test was used for a multigroup comparison of a single factor (e.g., effect of mouse genotype on TSH levels). An unpaired two-tailed Student’s *t*-test was used for the comparison of two independent groups. All statistical analyses were calculated with GraphPad Prism, version 10 (GraphPad Software, Inc., Boston, MA, USA). All data are expressed as mean ± SD. Differences of *p* < 0.05 were considered significant.

## Data Availability

All data are contained within the article, with primary data available upon request from the corresponding authors.

## Supplementary Materials

The following supporting information can be downloaded at: https://www.mdpi.com/article/10.3390/ijms26157166/s1.

## Abbreviations

Cas9	CRISPR-associated protein 9
ChEL	Cholinesterase-like domain
DAPI	40,6-diamidino-2-phenylindole
Tg	Thyroglobulin protein
TG	Thyroglobulin gene
T4	Thyroxine
T3	Triiodothyronine
TSH	Thyroid stimulating hormone
