# Supplementary material for: A Genetically-Engineered Thyroid Gland Built for Selective Triiodothyronine Secretion
Source: Int J Mol Sci. 2025 Jul 24;26(15):7166. doi: 10.3390/ijms26157166 (PMC12346460; doi:10.3390/ijms26157166)
Supplement: Supplementary file 1 [file ijms-26-07166-s001.zip › ijms-3668519-supplementary.pdf]

Supplemental Materials

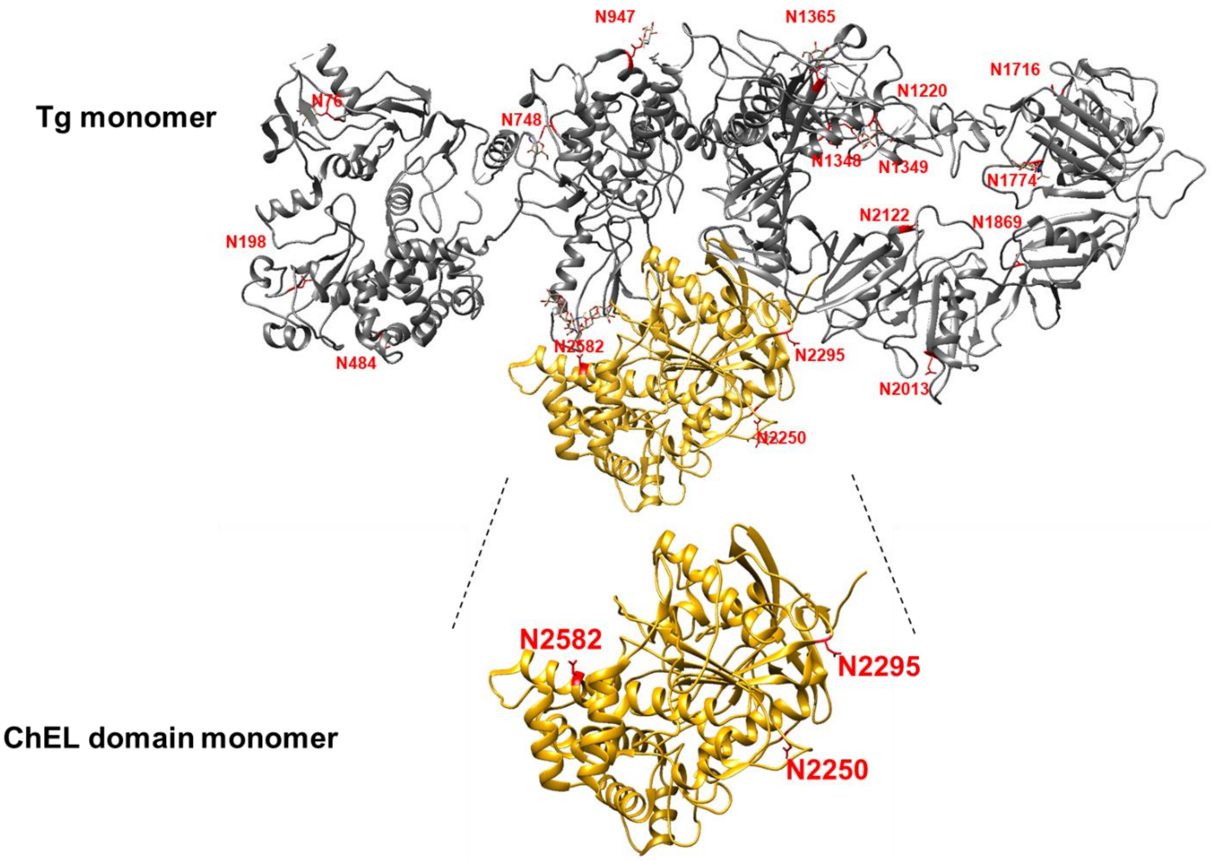

Figure S1. N-glycosylation within the ChEL domain of thyroglobulin (Tg). Structural representation of human (h) Tg monomer with its upstream regions colored grey and ChEL domain highlighted in yellow color (PDB 7B75), prepared using UCSF ChimeraX as described previously [1]**.** Asp residues at N-linked glycosylation sites, identified by LC-MS, are colored red. N2250, 2295 and 2582 in hTg-ChEL domain are located at equivalent amino acidic positions in mouse Tg-ChEL: N2249, N2294, and N2581 (GlyGen O08710).

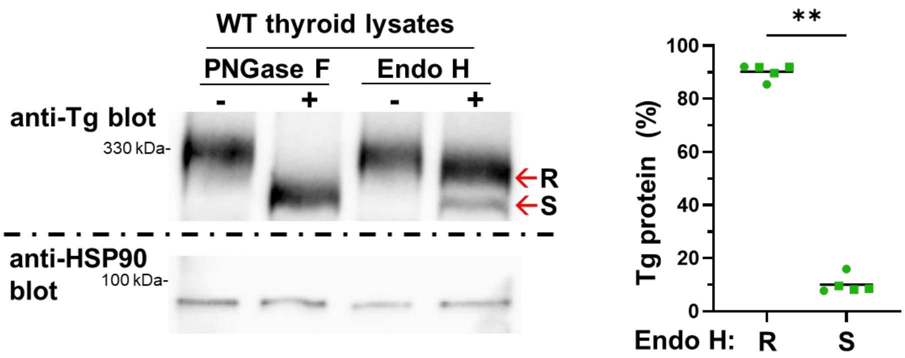

Figure S2. Confirmation of full-length Tg trafficking in vivo. SDS-polyacrylamide gel electrophoresis of thyroid homogenates from female wild-type (WT) mice previously treated (+) or untreated (-) with PNGase F or endoglycosidase H (Endo H), followed by immunoblotting with a mAb anti-Tg and a mAb anti-HSP90 as indicated. Red arrows point at Endo H resistant (R) or Endo H sensitive (S) content within Tg. Graph shows quantitation of Endo H R content within as well as the Endo H S content in Tg, from five independent thyroid homogenates from WT mice; mean ± SD; **, $p<0.01$ (unpaired two-tailed Student's t-test). Each dot represents an individual animal (circles represent females, squares represent males).

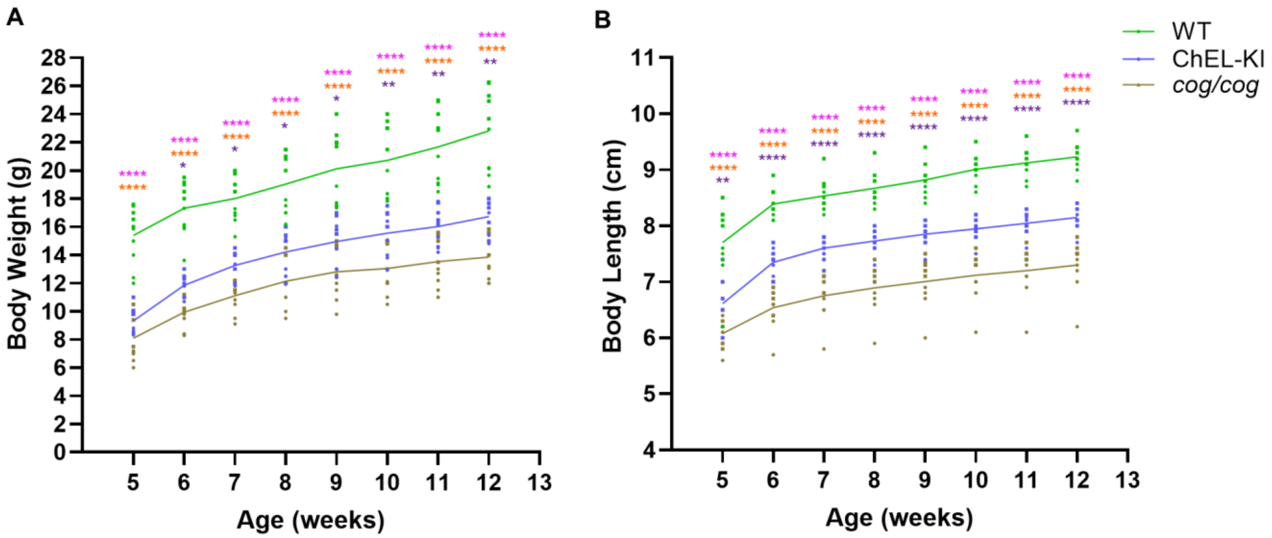

Figure S3. Body weight and body length of 5- to 12-week-old ChEL-KI mice. (**A**) Body weight of ChEL-KI mice, hypothyroid *cog/cog* animals, and euthyroid (wild-type, WT) controls. (**B**) Body length of ChEL-KI mice, hypothyroid *cog/cog* animals and WT controls. Each dot represents an individual animal (circles represent females, squares represent males), *n* = 10 mice per group, line is connecting mean values for each genotype, *P < 0.05, **P < 0.01, ****P < 0.0001 (two-way ANOVA with Tukey's comparison sample test). Graphs show significant differences between WT vs. ChEL-KI in pink color, WT vs. *cog/cog* in orange color, and ChEL-KI vs. *cog/cog* in violet color.

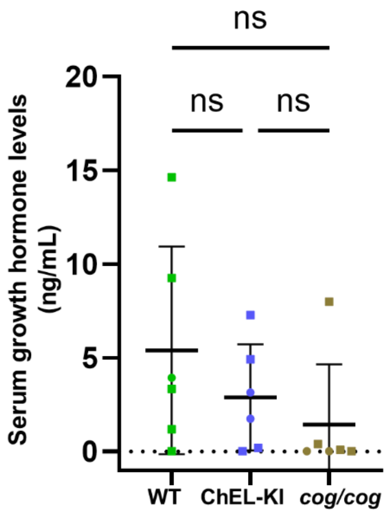

Figure S4. Serum growth hormone levels in ChEL-KI mice. Levels of serum growth hormone in 3-month-old ChEL-KI mice, hypothyroid *cog/cog* controls, and euthyroid (WT) controls, as determined by ELISA. Graph shows mean ± SD; n = 6 mice per group; ns, not significant (one-way ANOVA with Tukey's post hoc test). Each dot represents an individual animal (circles represent females, squares represent males).

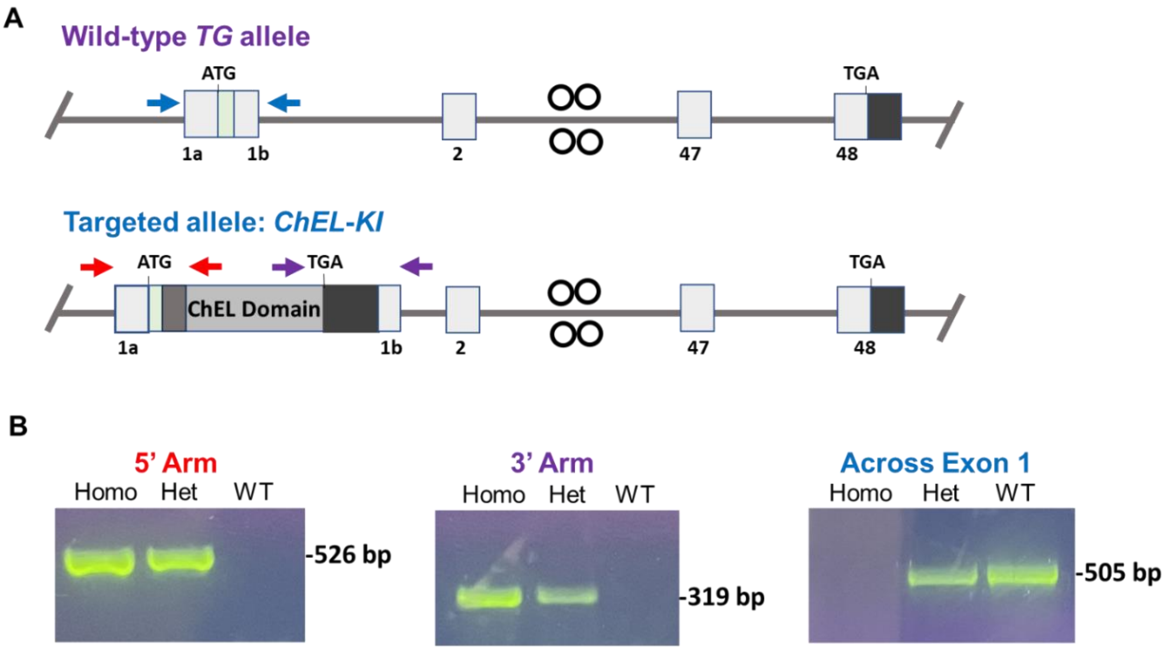

Figure S5. Genotyping of ChEL-KI mice through end-point PCR. (**A**) Schematic representation of the wild-type (WT) and ChEL-KI alleles as well as the localization of the PCR primer sets utilized for genotyping. Primer sets indicated by blue arrows amplify DNA across exon 1 in the WT allele. Primer sets indicated by red, and violet arrows amplify DNA across the 5′ arm and the 3′ arm of the ChEL-KI allele, respectively. (**B**) Discrimination of WT, heterozygous (Het) and homozygous (Homo) ChEL-KI genotypes. PCR products were size separated by electrophoresis on a 1.5% agarose gel. The expected amplicon size is 526-bp for 5′ arm of the ChEL-KI allele, 319-bp for 3′ arm of the ChEL-KI allele, and 505-bp for the WT allele.

Table S1. Primers used for genotyping of ChEL-KI mice. List of DNA sequences of different primer sets (with amplicon sizes) used to amplify target regions of the ChEL-KI allele and the wild-type (WT) mouse *TG* gene. F, forward. R, reverse.

PCR reaction name	Primer name	Primer sequence (5'-3')	PCR product size
5' Arm	F1	ATAGACTGGAGTGGTCACCCTACTGA	WT: 0bp<br>ChEL-KI: 526bp
	R1	CTTGGTAGCATCCCATGAACCTG	
3' Arm	F2	GCATCGCATTGTCTGAGTAGGTG	WT: 0bp<br>ChEL-KI: 319bp
	R2	ACTCTCAATATCCAGCTCTGGGACC	
Across Exon 1	F3	ATAGACTGGAGTGGTCACCCTACTGA	WT: 505bp<br>ChEL-KI: 2503bp (does not amplify using a regular PCR)
	R3	ACTCTCAATATCCAGCTCTGGGACC	

References

[1] Citterio CE, Kim K, Rajesh B, Pena K, Clarke OB, Arvan P. Structural features of thyroglobulin linked to protein trafficking. Protein Sci 2023;32:e4784. https://doi.org/10.1002/pro.4784.
